# Preparation and Characterization of Flame-Retarded Poly(butylene terephthalate)/Poly(ethylene terephthalate) Blends: Effect of Content and Type of Flame Retardant

**DOI:** 10.3390/polym11111784

**Published:** 2019-10-31

**Authors:** Weizhou Zhang, Cheng Zheng, Yuhui Zhang, Weihong Guo

**Affiliations:** Polymer Processing Lab, Shanghai Key Laboratory of Advanced Polymeric Materials, Key Laboratory for Ultrafine Materials of Ministry of Education, School of Materials Science and Engineering, East China University of Science and Technology, Shanghai 200237, China

**Keywords:** flame retardant, PBT, PET, DOPO, expandable graphite

## Abstract

A flame retardant named TAD was synthesized by the reaction of 9,10-Dihydro-9-oxa-10-phosphaphenanthrene-10-oxide and triallyl isocyanurate at first. Then, novel flameretarded materials based on PBT and PET resin were formulated via melt blending with TAD, expandable graphite (EG), and a mixture of both. The effect of flame retardant type and TAD content on the flame behavior of PBT/PET blend was carefully investigated. TAD contributed towards higher LOI value and better UL-94 performance than EG. However, the best V-0 rating in the UL-94 test was achieved by the incorporation of TAD/EG mixture into the resin matrix. TAD/EG combination exhibited clear synergistic effect on both reducing the flaming intensity and increasing the residual char layer, as confirmed by cone calorimeter tests and TGA results. SEM images combined with XPS analysis revealed that expansion and migration of EG locked the P-containing radicals from decomposing TAD into the condensed phase, which led to the formation of compact and continuous char layers. All the results in our studies demonstrate that incorporation of TAD with a charring agent EG is an effective and promising technique to develop flame-retarded PBT/PET material, which has high potential for applications in the areas of electronic devices, household products, and automotive parts.

## 1. Introduction

Poly(butylene terephthalate) (PBT) and poly(ethylene terephthalate) (PET) are commonly considered as two of the most important engineering polyesters in industry [1,2]. PBT shows a wide range of applications in electronic and automotive products due to its rapid crystallization rate, solvent resistance, good dimensional stability, excellent electrical properties, and good processability [3,4]. Unlike PBT, which is primarily used in injection molding parts, the main application areas of PET are fibers, films, and containers for packaging [5,6,7]. PET has higher heat-deflection temperature and stiffness compared to PBT, while PBT demonstrates advantages in crystallization rate, processing, and dimensional stability [8,9]. Blending two or more polymers has been shown as a simple, effective, and low-cost approach to obtain a novel composite with integrated and potential enhanced properties without clearly sacrificing their advantages [10,11,12]. Thus, development of PBT/PET blends has attracted a significant amount of attention from researchers and industry [13,14,15,16]. Blending PBT with PET achieves a product with high electrical insulation properties and good mechanical properties due to the synergistic effect of these two polyesters in the crystallization process [17]. Besides, the crystallization behavior of PET was significantly enhanced by blending with PBT, resulting in a lower glass transition temperature and melting temperature [18]. However, an additional excellent flame-retardant property is required for utilization in the fields of electronic devices, household products, and automotive parts [19,20]. Therefore, it is critical to improve the flame retardant performance of the PBT/PET blend because both PBT and PET are highly flammable with dripping behavior during combustion. However, studies on flame retardant PBT/PET blends have barely been reported till date.

Blending polymer matrix with additive flame retardants has proven as a promising and effective way to improve the anti-flaming performance of the polyester [21]. Among these flame retardants, the 9,10-Dihydro-9-oxa-10-phosphaphenanthrene-10-oxide (DOPO) and its derivatives are widely added into polyesters because they are halogen-free and have high thermal stability and good anti-oxidation ability [22,23,24]. They act as free radical releasing agents, providing P-containing fragments such as PO_2_^.^ and PO^.^ to quench the free radicals from degraded resin matrix and terminate the chain reaction in the gas phase [25,26]. Moreover, to further increase the flame retardant efficiency of DOPO, research efforts have been devoted to design and synthesize novel DOPO derivative by combining DOPO with other flame-retardant agents. Especially, after reacting with triazine-based flame retardants, such as melamine cyanurate (MCA) and melamine polyphosphate (MPP), the flame-retardant effect of DOPO in the gas phase can be significantly enhanced owing to the inert, incombustible Ncontaining gas released by triazine-based flame retardants under heating [27,28]. Recently, an emerging flame retardant named TAD was synthesized by Tang et al. [29,30] by combining DOPO with triallyl isocyanurate (TAIC) exhibiting high flame retardant efficiency in the epoxy resin. This TAD was found to act mainly in gas phase, with additional slight charring effect, which might be less effective for anti-dripping of PBT/PET blends during combustion. On the other hand, expandable graphite (EG), as an economic and well-performed charring additive, is widely applied to develop a number of fire-retardant applications [31,32,33]. It is an intercalated graphite compound whereby oxidants such as sulfuric acid and potassium permanganate are inserted between the carbon layers of graphite. When exposed to a heat source, EG can expand in the perpendicular direction and generate a vermicular structured layer to protect the matrix from heat flux penetrating inside and retard the further decomposition of polymer chain [34]. However, if EG is used as the only fire retardant in the polymer, its efficiency is low and limited [35]. Consequently, a new fire retardant system based on combination of TAD and EG was designed in this article in order to overcome the disadvantages of these two fire retardants and obtain enhanced synergistic anti-flaming effect. Hence, the aim of this work is to develop novel materials with excellent fire retardant performance based on the PBT/PET blends and the fire retardant system of TAD and EG. First, the flame retardant TAD was carefully synthesized. Next, fire retardant materials were formulated by blending TAD or TAD/EG with a toughing PBT/PET blend reported in our previous work [9].To the best of our knowledge, the research work about introducing this novel flame retardant system based on TAD and EG to improve the anti-flaming property polyesters is rarely reported. The effect of TAD loading and addition of EG on the flame-retardant performance, thermal properties, and flaming behavior of flame-retarded PBT/PET blends were investigated using different techniquessuch as limited oxygen index (LOI), UL-94 vertical burning test, cone calorimeter tests, and thermogravimetric analysis (TGA). Simultaneously, the flame-retardant mechanism of TAD and TAD/EG combination on PBT/PET blend was also explored by scanning electronic microscopy (SEM) and X-ray photoelectron spectroscopy (XPS).

## 2. Materials and Methods

### 2.1. Materials

PBT (TH6100) and PET (TH105) were supplied by Blueridge Tunhe Chemical Industry Co., Ltd. (Xinjiang, China), with an intrinsic viscosity of 1.001 dl g^−1^ and 0.765 dl g^−1^ respectively. POE-g-GMA (GPOE) grafted with 0.7 wt% glycidyl methacrylate was purchased from Ningbo Nengzhiguang New Materials Technology Co., Ltd. (Zhejiang, China). Surlyn 8920, the nucleating agent, was kindly provided by DuPont (Shanghai, China). TAIC and DOPO were purchased from Aaladin (Shanghai, China), while expandable graphite (EG) (Kp80) was purchased from Aoyu Graphite Company (Shanghai, China).

### 2.2. Synthesis of TAD

**Figure 1:**
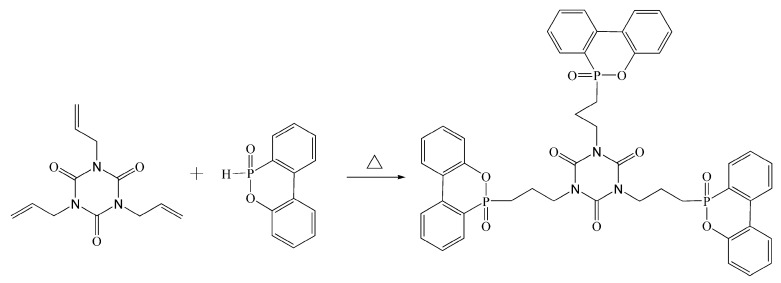
Synthesis route of TAD.

The TAD was prepared using a one-step synthesis method, as previously described [29]. DOPO (324 g, 1.50 mol) was firstly melted at 145 °C with mechanical stirring in a three-neck flask. Then, TAIC (124.5 g, 0.50 mol) was introduced into the melt DOPO at the addition rate of 12.45 g per 5 min. The temperature of reaction system was then heated to 155 °C with mechanical stirring for another 2 h. The final TAD powder was collected from the cooled and ground reacted product at room temperature. The reaction routine is shown in Figure 1.

### 2.3. Preparation of Flame-Retardant PBT/PET Blend

All the PBT and PET pellets, POE-g-GMA, nucleating agent, and fire retardants were dried in a ventilated oven before processing to avoid possible moisture degradation reactions. The detailed formulations of different samples are summarized in Table 1. In the resin matrix, the PBT/PET/GPOE/Surlyn 8920 ratio is fixed as 40/60/20/0.3. Different combinations of fire retardants were mixed evenly with matrix resins and other additives before extrusion. Then, the mixture was introduced into a corotating twin-screw extruder (TSE-35A, Nanjing Ruiya Co., Ltd., China). Notably, the length to diameter ratio of the screw was 48, the diameter of the screw was 35 mm, and the temperature profiles of the barrel were 40-160-180-200-220-230-240-250-245 °C from the hopper to the die. The extruded rods were dried at 80 °C for 6 h and then hot pressed (10 MPa, 5 min, 250 °C) to obtain suitable testing bars for further characterization.

### 2.4. Characterization

The limited oxygen index was tested by a HC-2C oxygen index meter (Nanjing Shangyuan Analysis Instrument Company, China) according to ISO 4589-1984, and the specimens used for the test were 130 mm × 6.5 mm × 3 mm in dimension.

The UL-94 vertical burning tests were performed on a CZF-2 instrument (Nanjing Jiangning Analytical Instrument Factory, China). The dimensions of the sample were 130 mm × 13 mm × 3 mm.

The thermal combustion properties of samples were measured with a cone calorimeter (FTT, East Grinstead, UK) as per ISO5660 at an external heat flux of 50 kW/m^2^. The dimension of the samples was 100 mm × 100 mm × 3 mm.

The thermogravimetric analysis was performed on a STA409 PC/PG machine (Netzsch, Bavaria, Germany). The sample masses ranging from 2 to 3 mg were heated from room temperature to 600 °C at the rate of 20 °C/min under a nitrogen atmosphere.

Scanning electronic microscopy (SEM) was performed using a S-3400N instrument (Hitachi, Tokyo, Japan) to observe the surface morphology of the char layer formed from specimens after cone calorimeter testing.

The elemental analysis of the residual char from samples after cone calorimeter testing was performed on a 1/AXIS UltraDLD X-ray photoelectron spectroscopy (Kratos, Kyoto, Japan). Residual chars were sufficiently ground and mixed before analysis.

The tensile, flexural, and impact property of all samples were tested on a Universal Testing Machine (MTS, Eden Prairie, MN, USA). At least 5 specimens of each formulation were tested, and the average value was calculated.

## 3. Results and Discussion

### 3.1. Flame-Retardant Performance

The LOI and UL-94 vertical tests were performed to determine the flame performance of PBT/PET blend and flame-retarded PBT/PET materials, and the results are summarized in Table 1. Without the addition of fire retardants, the resin matrix displayed an extremely low 22.0 LOI value. In addition, the test bar burned continuously, accompanied with flammable dripping during UL-94 test. After the addition of low 4% TAD, the LOI value of PBT/PET blend significantly increased to 25.2; meanwhile, the UL-94 rating still remained as no rating (NR). The LOI values of samples gradually increased with further addition of TAD in the PBT/PET blends. Besides, the UL-94 performance of PBT/PET blend was also enhanced with the TAD content. Similar findings were also observed in the EP thermosets with TAD. When the TAD content reached 12 wt%, the LOI value of the sample reached 28.4 and passed UL-94 V-1 rating. When the TAD loading was increased to 16% in the composite, the LOI value showed a slight decrease, but the UL-94 rating remained unchanged. Blend only with 12 wt% EG had a LOI value of 25.8, which is lower than the sample only containing 12 wt% TAD, indicating that the addition of equivalent TAD contributed towards more clear effect on increasing LOI value of PBT/PET blend. Notably, after the incorporation of 6 wt% TAD and 6 wt% EG into the matrix resin, the blend reached the highest LOI value of 29.2 and passed UL-94 V-0 rating. These results imply that using TAD and EG mixture leads to more significant flame-retardant effect on the PBT/PET blend compared to neat TAD.

### 3.2. Cone Calorimeter Test

**Figure 2:**
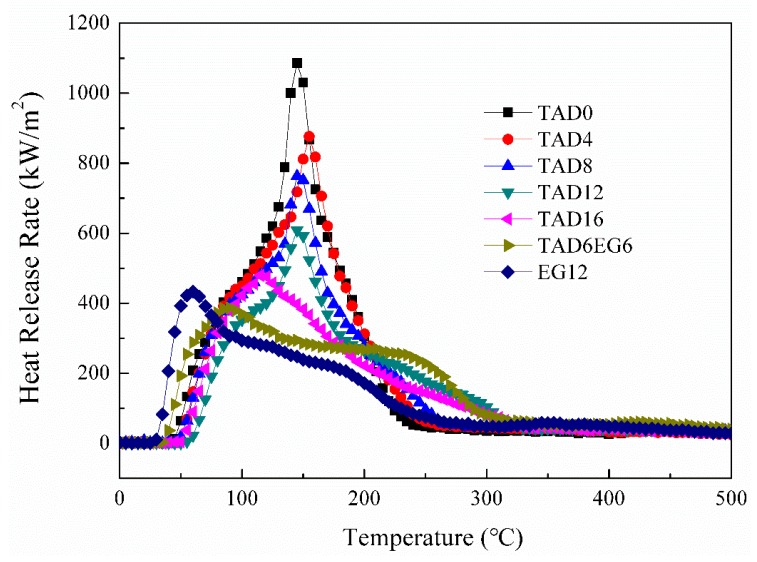
Heat release rate (HRR) curves of PBT/PET blend and different flame-retardant PBT/PET materials.

Cone calorimeter test was employed to characterize thermal combustion behavior of PBT/PET blend and flame-retarded PBT/PET blends. Figure 2 displays the heat release rate (HRR) curves of the PBT/PET blend and different flame-retarded PBT/PET materials. Table 2 summarizes the partial characteristic parameters obtained from cone calorimeter test, such as peak of heat release rate (PHRR), total heat release (THR), average of effective heat of combustion (mean-EHC), average CO_2_ yield (mean-CO_2_Y), and total smoke release (TSR).

The PBT/PET blend without flame retardant had an extremely high PHRR value of 1087.7 kW/m^2^. After incorporation of TAD, the PHRR value gradually decreased with TAD content in the blend. The parameter PHRR is usually employed to assess the flammability of materials; thus, the results prove that TAD can effectively inhibit the combustion intensity of PBT/PET blend, which is also in agreement with the LOI results. The mean-EHC values of PBT/PET blends containing TAD were also reduced compared to blend without TAD, indicating that the amount of fuels was decreased. The reduction of mean-EHC is due to the quenching effect of the decomposed TAD fragments, which terminated the combustion free radical chain reaction and decreased the amounts of fuels. THR is commonly used to evaluate the fire safety of the materials in a real fire. As shown in Table 2, TAD contributed to lower THR value of PBT/PET blends especially at high addition amount, implying that the fireretardant effect of TAD is more prominent at high loadings, which is in agreement with the UL-94 results. Compared with TAD12, the sample TAD6EG6 with TAD and EG mixture showed similar THR value, but significantly lower PHRR and mean-EHC value, which may partly explain why TAD6EG6 passed UL-94 V-0 rating but TAD12 could only reach V-1 rating. Regarding to sample EG12 with 12 wt% EG, the HRR curve shifts a bit left compared with the curve of other samples with TAD, indicating EG is easy to be activated on fire [36]. It is interesting that EG12 exhibits lower PHRR and THR compared with TAD12, while it has worse UL-94 performance than TAD12. This phenomena is due to that EG can not act like TAD to eliminate the free radical groups formed during combustion and release inert gas to dilute flammable gas, resulting in long flaming time over 30 s (NR rating in UL-94). Mean-CO_2_Y value was also decreased after the addition of TAD in the PBT/PET blend, which demonstrates that the resin matrix combusted less sufficiently than PBT/PET blend without adding fire retardants. This is strong evidence that TAD can effectively hinder the combustion of volatiles in the gas phase during fire, resulting in less CO_2_. Furthermore, the TSR value of PBT/PET blends gradually increased with TAD loading, indicating formation of more residue char instead of fuels during combustion. Notably, PBT/PET blend with the mixture of TAD and EG (TAD6EG6) had further lower value of mean-CO_2_Y but higher value of TSR compared to that only with TAD (TAD12) or EG (EG12), suggesting that TAD and EG possessed a clear synergistic effect on both inhibiting the burning intensity and promoting char formation.

### 3.3. Thermal Stability

**Figure 3:**
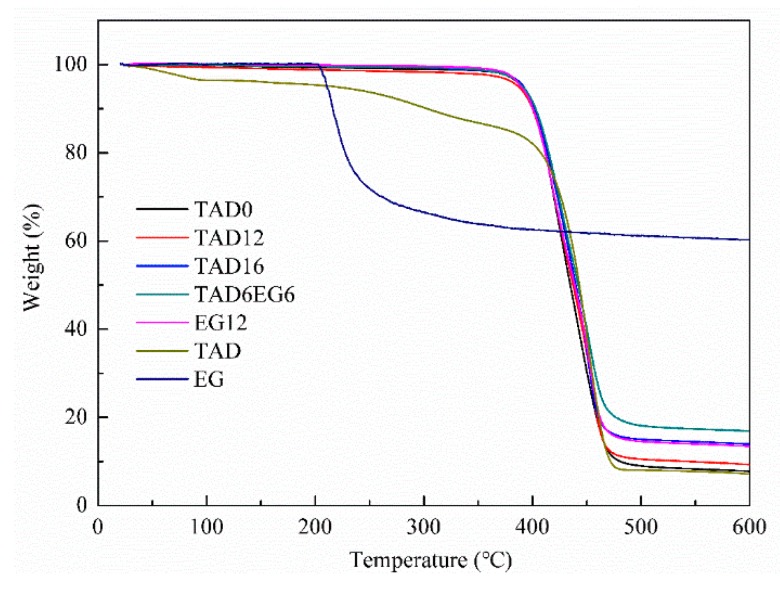
TGA curves of TAD0, TAD12, TAD16, TAD6EG6, EG12, TAD and EG under a nitrogen atmosphere.

The TGA curves of TAD0, TAD12, TAD16, TAD6EG6, EG12, and fire retardants TAD and EG under a nitrogen atmosphere are shown in Figure 3 and some typical data are collected in Table 3. The parameter T_5%_ refer to the temperature at which weight loss is 5%. The char residue (%) is the unburnt residue at 600 °C. The TGA curves of all the fire retarded materials showed similar shape, only with different solid residues at 600 °C. As per Table 3, there was no significant difference in T_5%_ between the neat resin matrix and fire-retardant composites, indicating that neither TAD nor EG exerted their flame resistance by inducing the decomposition of the resin matrix. The char residue of composites with TAD was clearly higher than that of the neat resin matrix. This is because the phosphaphenanthrene group of TAD would decompose to phosphoric acid or polyphosphoric acid compounds and promote the resin matrix to form more char residues during combustion. Comparing sample TAD12 and TAD6EG6, the combination of TAD and EG resulted in more char residues than TAD or EG individually, which reveals that combination of TAD and EG had a better charring effect on the PBT/PET blend. In order to determine whether interactions between TAD and EG occurred, the theoretical char residue was calculated from the experimental TGA data of TAD0, TAD, and EG, assuming no interactions. Theoretical char residue (C) was calculated according to the equation below:

C = P × 88% + T × 6% + E × 6%(1)

where P, T, E are char residue of PBT/PET blend without flame retardants, pure TAD, and neat EG, respectively. The result is 10.9% much lower than experimental data of TAD6EG6, confirming the synergistic flame-retardant effect between TAD and EG within the PBT/PET matrix and consequently increase the char residue.

### 3.4. Morphology

**Figure 4:**
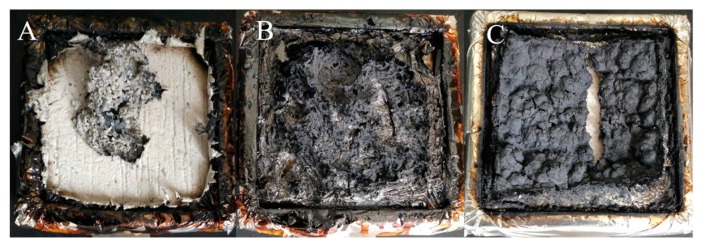
Digital images of char residue obtained from cone calorimeter test: (**A**) TAD0; (**B**) TAD12; and (**C**) TAD6EG6.

Figure 4 shows the digital images of PBT/PET blend and flame-retarded PBT/PET materials after cone calorimeter test. As shown, PBT/PET blend without anti-flame additive left small amount and thin char after burning, suggesting weak char forming ability (Figure 4A). After incorporation of TAD into the composite, more residue chars were formed (Figure 4B), corresponding to the TGA results. However, the status of this char, especially in the middle part, was fluffy and not compact. Similar morphology was also observed for TAD reinforced epoxy thermoset, which can be attributed to increased gas release under action of TAD during combustion. As shown in Figure 4C, combination of TAD and EG contributed to form more dense and compact char compared to neat TAD, demonstrating the synergistic effects between TAD and EG on char formation of PBT/PET blend.

**Figure 5:**
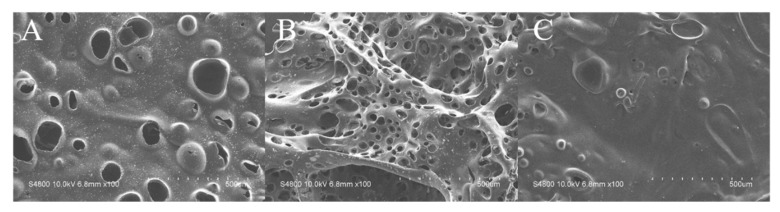
SEM images of char residue obtained from cone calorimeter test: (**A**) TAD0; (**B**) TAD12; and (**C**) TAD6EG6.

The microscopic morphologies of these residues were further characterized under SEM, and the results are shown in Figure 5. Large and open holes were clearly observed on the residual char of the neat resin matrix (Figure 5A), which were due to the volatilization of flammable gases during combustion. As for composite with 12 wt% TAD, the loose structure is clearly presented in Figure 5B. However, the porous structure cannot prevent the exchange of the fuel and oxygen or protect the matrix from the flame efficiently. Thus, this morphologycombined with cone calorimeter data demonstrates that the flame-retardant effect of TAD in gas phase was stronger than that in the condensed-phase. Figure 5C shows a compact and continuous surface of the char layer from composite with 6 wt% TAD and 6 wt% EG, implying that TAD and EG interacted in the condensed phase and resulted in the formation of well-sealed char layer, which protect the matrix from the penetration of heat flux and retards further decomposition of the resin matrix. This phenomenon may explain the reason behind better UL-94 rating of sample TAD6EG6 than sample TAD12.

### 3.5. Flame-Retardant Mechanism

To further investigate the synergistic flame-retardant effect of TAD and EG, XPS was used to analyze the elemental contents of the residual chars from cone calorimeter tests, and the results are summarized in Table 4. The relative C content of residual char from PBT/PET blend in combination with TAD and EG was higher than that of composite with only TAD owing to the thermal stability of EG at high temperatures. Besides, combination of flame retardants contributed to more relative P content remaining in the char residue as that 0.25% P per 1% TAD was reserved in the char of TAD6EG6 but only around 0.18% P per 1% TAD was kept in the char of TAD12. The remaining N content in the TAD6EG6 was 0.14% N per 1% TAD, which is slightly lower than that of TAD12 (0.16% N per 1% TAD). According to the previous research work [24,29,37,38], the flame-retardant effect of TAD was due to its decomposition products during combustion: (i) P-containing free radicals which can quench the free radicals from degraded resin matrix, and terminate the chain reaction in the gas phase, and (ii) incombustible N-containing gas which diluted the flammable gases released from the resin matrix. TAD acts weak in the condensed phase due to that most of the phosphorus is released to the gas phase, and only minor charring activity is remained [30]. Hence, the mechanism for the synergistic effect of TAD and EG can be concluded as follows. During combustion, EG initially expanded and migrated on the resin matrix, locked more P-containing fragments from decomposed TAD into the condensed phase to form a compact and continuous char layer with significant enhanced barrier effect. The amount of P-containing fragments in the gas phase decreased; however, the release of inert N-containing gas was not clearly negatively impacted. Consequently, the overall flame-retardant performance of the combined TAD and EG on the PBT/PET blend was better than that of neat TAD.

### 3.6. Mechanical Properties

To study the influence of adding flame retardants on the mechanical property of PBT/PET blend, mechanical testing was performed. The results are summarized in Table 5. Without adding flame retardants, the specific PBT/PET blend developed by us shows high impact property with good balance between toughness and stiffness. The bending and tensile property of PBT/PET blend are slightly enhanced by adding 4 wt% TAD, which is due to TAD acts as filler at low addition amount. After incorporation of 12 wt% TAD, decrease of stiffness was clearly observed, might owing to poor interface interaction between TAD and polymer matrix or aggregation of fire retardants. In addition, adding EG contributes to further decrease of flexural and tensile performance compared to TAD at the same addition amount of 12 wt%. The values of bending modulus, bending strength, and tensile strength of PBT/PET blend with combination of TAD and EG were in-between those of TAD- and EG-based PBT/PET materials. The clear degeneration was observed in impact property after adding flame retardants. Notably, when adding 6 wt% EG and 6 wt% TAD in PBT/PET blend, the notched impact strength is 15.1 kJ/m^2^, only 28.5% of the value of PBT/PET blend without flame retardants. However, considering the additional excellent anti-flaming performance, TAD6EG6 with overall relatively considerable mechanical property, still has high potential for applications in the areas of electronic devices, household products, and automotive part.

## 4. Conclusions

Novel flame-retardant materials were successfully developed based on the PBT/PET blend and TAD or TAD/EG combination. The effect of TAD loading and addition of EG on the flame-retardant property, flame behavior, and thermal stability of the resulting materials were explored. TAD contributed to higher LOI value than EG. However, PBT/PET blend with EG/TAD combination exhibited better UL-94 performance compared to that with only TAD. Cone calorimeter test combined with TGA confirmed that TAD/EG combination possessed a clear synergistic effect on both inhibiting the burning intensity and promoting the char formation. SEM images and XPS analysis revealed that the synergistic flame-retardant effect was due to the expansion and migration of EG on the resin matrix which locked more P-containing fragments from decomposed TAD into the condensed phase to form a compact and continuous char layer with significantly enhanced barrier effect, without much loss of the quenching effect of TAD in the gas phase. All these results clearly demonstrated that the incorporation of TAD with a charring agent EG is an effective and promising method to enhance the anti-flame properties of PBT/PET blend, although degeneration was observed in mechanical property compared to neat PBT/PET blend. The resultant material exhibited high potential for applications in the areas of electronic devices, household products, and automotive parts.

## Figures and Tables

**Table 1:** Sample codes, formulations, LOI, and UL-94 test results of PBT/PET blend and flame-retarded PBT/PET materials.

Samples	Resin Matrix	TAD (wt%)	EG (wt%)	TAD/EG	LOI	UL-94
TAD0	100	0	0	/	22.0	NR
TAD4	96	4	0	/	25.2	NR
TAD8	92	8	0	/	26.9	V-2
TAD12	88	12	0	/	28.4	V-1
TAD16	84	16	0	/	28.1	V-1
TAD6EG6	88	6	6	1/1	29.2	V-0
EG12	88	0	12	/	25.8	NR

**Table 2:** Cone calorimeter test results of PBT/PET blend and flame-retardant PBT/PET materials.

Sample	PHRR (kW/m^2^)	Mean-EHC (MJ/kg)	THR (MJ/m^2^)	Mean-CO_2_Y(kg/kg)	TSR (m^2^/m^2^)
TAD0	1087.7	17.1	92.0	1.48	1530.9
TAD4	885.1	8.6	94.2	0.91	1853.5
TAD8	777.9	8.9	91.0	0.92	1944.3
TAD12	616.7	10.4	84.3	0.99	2105.0
TAD16	486.7	10.6	72.1	1.05	2236.5
TAD6EG6	390.4	7.8	84.7	0.67	2622.9
EG12	435.2	10.2	78.3	0.83	2412.5

**Table 3:** Thermal parameters of PBT/PET blend and flame-retardant PBT/PET blends.

Sample	T_5%_ (°C)	Char Residue (%)
TAD0	390	7.8
TAD12	389	9.3
TAD16	391	13.9
TAD6EG6	390	16.9
EG12	390	14.4
TAD	218	7.2
EG	211	60.2

**Table 4:** Elemental content of char residues from cone calorimeter test obtained via XPS.

Sample	C (%)	N (%)	O (%)	P (%)
TAD12	81.2	1.9	14.9	2.1
TAD6EG6	90.5	0.8	7.2	1.5

**Table 5:** Mechanical properties of PBT/PET blend and flame-retarded PBT/PET materials.

Samples	Notched Impact Strength (kJ/m^2^)	Bending Modulus (MPa)	Bending Strength (MPa)	Tensile Strength (MPa)
TAD0	53.1± 4.7	1454 ± 12	47.5 ± 1.8	36.4 ± 0.5
TAD4	33.4 ± 2.4	1530 ± 25	50.6 ± 1.2	37.9 ± 0.6
TAD12	18.5 ± 1.6	1376 ± 16	44.0 ± 1.4	33.5 ± 1.6
TAD6EG6	15.1 ± 3.5	1312 ± 19	39.9 ± 1.7	30.7 ± 2.5
EG12	11.6 ± 4.9	1220 ± 36	36.7 ± 2.2	28.2 ± 2.9
